# A (Preliminary) Recipe for Obtaining a Testing Effect in Preschool Children: Two Critical Ingredients

**DOI:** 10.3389/fpsyg.2018.01446

**Published:** 2018-08-21

**Authors:** Oliver Kliegl, Magdalena Abel, Karl-Heinz T. Bäuml

**Affiliations:** Department of Experimental Psychology, Regensburg University, Regensburg, Germany

**Keywords:** episodic memory, young children, testing effect, retrieval practice, test format

## Abstract

The testing effect refers to the finding that retrieval of previously learned information improves retention of that information more than restudy practice does. While there is some evidence that the testing effect can already arise in preschool children when a particular experimental task is employed, it remains unclear whether, for this age group, the effect exists across a wider range of tasks. To examine the issue, the present experiments sought to determine the potential roles of retrieval-practice and final-test formats, and of immediate feedback during retrieval practice for the testing effect in preschoolers. Experiments 1 and 2 showed no testing effect in preschoolers when a free-recall task was applied during the final test, regardless of whether free recall (Experiment 1) or cued recall (Experiment 2) were conducted during retrieval practice. In contrast, if cued-recall tasks were used during both retrieval practice and the final test (Experiment 3), a reliable testing effect arose. Furthermore, the magnitude of the effect was dramatically enhanced when, in addition, immediate feedback was provided during retrieval practice (Experiment 4). The present findings suggest that cued-recall practice and test formats, as well as immediate feedback during practice, are crucial ingredients for obtaining the testing effect in preschoolers.

## 1. Introduction

A vast number of studies have shown that retrieval practice of previously studied information can improve long-term retention of that information quite dramatically, relative to repeated study of the information (e.g., Hogan and Kintsch, 1971; Roediger and Karpicke, 2006; McDaniel et al., 2007). This so-called testing effect has been demonstrated over a wide variety of materials and settings, in both lab-based and classroom studies (for reviews, see Roediger and Butler, 2011; Dunlosky et al., 2013). However, few studies have examined whether the effect can already arise in elementary school children, and there is even less research on whether preschool children can already benefit from retrieval practice. Indeed, there is only evidence from one recent study suggesting that retrieval practice can enhance preschool children's verbal learning. This study employed an experimental task in which (i) cued recall was used during retrieval practice and the later retention test, and (ii) immediate feedback via restudy was provided during retrieval practice (Fritz et al., 2007). Considering that one of the major goals of education is to teach children how to become effective learners, knowledge about if and when retrieval practice can become a beneficial tool to enhance learning and retention of information would appear essential. Therefore, the present study sought to determine whether the testing effect in preschool children is limited to the experimental task employed by Fritz et al. or whether the effect generalizes across a wider range of tasks.

Several studies so far have examined the testing effect in elementary school children (e.g., Gates, 1917; Bouwmeester and Verkoeijen, 2011; Lipowski et al., 2014; Karpicke et al., 2016). The results from these studies suggest that, in general, children at that age can benefit from retrieval-practice opportunities. For instance, Lipowski et al. (2014) showed first and third graders pictures of objects that were either studied five times in succession (SSSSS) in the restudy condition, or were studied three times and orally tested after the first and second study cycle in the test-plus-restudy condition (STSTS). A free-recall test that was conducted 5 min after practice revealed a reliable testing effect, with no difference in the size of the effect between age groups. The findings suggest that the testing effect can already be obtained in younger elementary school children, with no major developmental trend during these school years.

To date, only a single study has addressed the issue of whether preschoolers' verbal learning can also benefit from retrieval practice. In this study, Fritz et al. (2007) had children learn the names of seven toy pigs, such as *Tinker* for a pink pig, by means of either 4 expanding restudy-practice cycles or 4 expanding retrieval-practice cycles. During each restudy cycle, the children were presented with each of the toys and the experimenter provided the correct name, whereas, during each retrieval-practice cycle, the children were shown each of the toys, but were asked to say out loud its correct name. Critically, immediate feedback was provided during retrieval-practice cycles because, regardless of whether or not the child was able to recall a toys' name on a given trial, the experimenter gave the correct name at the end of the trial. On a later cued-recall test in which the children were again shown each of the toy animals and asked to say their respective names, recall of the toys' names turned out to be significantly improved with prior retrieval practice compared to prior restudy practice, thus suggesting that preschool children can already show the testing effect.

In a sense, however, the practice and test conditions in the Fritz et al. (2007) study may constitute a best-case scenario. In this study, (i) cued recall was used during both retrieval practice and the final test, and (ii) immediate feedback was provided during retrieval practice. In adults, cued-recall practice formats are often complemented with immediate feedback, and this type of practice has been found to generally lead to a more pronounced testing effect than, for instance, free-recall formats without immediate feedback (Pashler et al., 2005; Kang et al., 2007; Rowland, 2014). If cued-recall formats, as well as the presence of immediate feedback during practice were similarly beneficial for the testing effect in preschoolers as in young adults, then the testing effect in preschoolers may be reduced, if not eliminated, with more demanding test formats, like free-recall tasks without immediate feedback during practice. The present study examined the issue.

The results of 4 experiments are reported designed to examine whether, in preschool children, the presence of the testing effect depends on the type of task employed during retrieval practice and test. In Experiment 1, we employed a version of the testing-effect task that was very similar to the task that Lipowski et al. (2014) had used to demonstrate the testing effect in elementary school children. In this task, children's memory of a previously studied and practiced list of items was assessed, with practice either consisting of repeated test and study cycles, or repeated study cycles only. Importantly, like in the Lipowski et al. study, but different from the Fritz et al. (2007) study, free-recall practice and test formats were used, and there was no immediate feedback during practice. Because such formats typically yield a reduced testing effect in young adults, it was expected that the effect might be small, if existent at all, in preschool children. Experiment 2 was similar to Experiment 1, but a cued-recall practice format, instead of the more demanding free-recall format was used, to examine whether increased success rates during practice might boost the testing effect. In Experiment 3, cued-recall practice and cued-recall test formats were used to determine how a less demanding final test would affect the testing effect in preschoolers. Finally, Experiment 4 more closely followed the procedural details of the Fritz et al. (2007) study, and, unlike Experiments 1–3, provided immediate feedback during retrieval practice (for an overview of the differences between the single experiments, see Table 1).

**Table 1:** Overview of the critical differences between Experiments 1–4 regarding the retrieval-practice and final-test formats, and the presence of immediate feedback during retrieval practice.

	**Retrieval-practice format**	**Final-test format**	**Immediate feedback**
Experiment 1	Free recall	Free recall	No
Experiment 2	Cued recall	Free recall	No
Experiment 3	Cued recall	Cued recall	No
Experiment 4	Cued recall	Cued recall	Yes

## 2. Experiment 1

Experiment 1 examined whether preschool children can benefit from retrieval practice when free-recall practice and test formats are used, and when no immediate feedback is provided during practice. We employed a version of the testing-effect task that, with respect to most procedural details and the study materials employed, was very similar to the task Lipowski et al. (2014) had used. In this task, preschool children studied a list of items, which was then either repeatedly retrieval practiced and restudied or repeatedly restudied only. After a delay, the children were asked to recall all study items in any order they wished. The results of the experiment will indicate whether the findings of Fritz et al. (2007) for preschoolers, which arose under conditions in which retrieval is not particularly demanding, generalize to more difficult retrieval conditions.

### 2.1. Method

#### 2.1.1. Participants

Thirty-two preschoolers (4–6 years; *M* = 5.4, *SD* = 0.7, 18 female) took part in the experiment. The children were recruited from two kindergartens in Regensburg, Germany. All children were tested individually in a quiet room. This study was carried out in accordance with the recommendations of the German Association of Psychology(DGPs). All parents gave written informed consent in accordance with the Declaration of Helsinki.

#### 2.1.2. Material

Two sets of study items were employed. Following Lipowski et al. (2014), each set consisted of pictures from four taxonomic categories (e.g., animals, fruits), with four objects in each category (e.g., *elephant, apple*). Participants were asked to study one set of items in the restudy condition and the other set in the test-plus-restudy condition.

#### 2.1.3. Design and procedure

**Figure 1:**
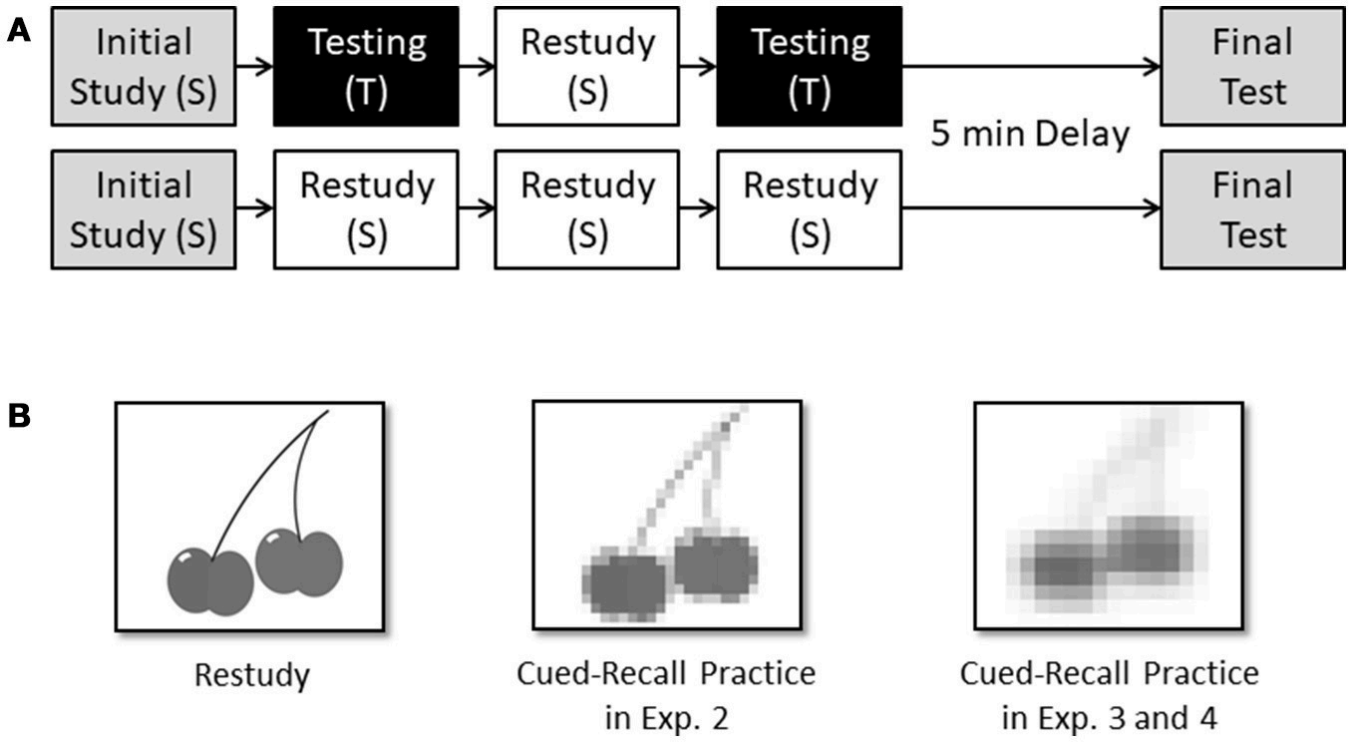
**(A)** Procedure employed in Experiments 1. In the test-plus-restudy condition (upper panel), initial study of a set of 16 pictures of objects was followed by two free-recall retrieval-practice cycles and one interspersed restudy cycle (STST). In the restudy condition (lower panel), initial study of a set of 16 pictures of objects was followed by three successive restudy cycles (SSSS). In both conditions, the four practice cycles were followed by a 5-min delay and a final free-recall test, in which children were asked to orally recall as many of the objects as they could. In Experiments 2–4, the same basic task was used, but with some critical variations (for details, see main text). **(B)** Types of practice in Experiments 1, 2, 3, and 4. During restudy cycles, children in Experiments 1–4 were re-exposed to each of the 16 pictures (left panel). During test-plus-restudy cycles, children in Experiment 1 were asked to orally recall as many of the study pictures as possible in a free recall test (not shown), while, in Experiments 2–4, they were shown pixelated versions of each of the study pictures and were asked to recall the name of the object. In Experiment 2, the children were cued with slightly less pixelated versions of the pictures (middle panel) than in Experiments 3 and 4 (right panel). In Experiments 3 and 4, pixelated versions of the pictures like the example depicted in the right panel were also used during the final test.

Type of practice was varied within participants. Following initial study, participants performed either repeated retrieval practice and restudy cycles (test-plus-restudy condition) or repeated restudy cycles only (restudy condition; see Figure 1A). Order of conditions and assignment of study sets to conditions were counterbalanced. In the restudy condition, participants were shown a set of items in four successive study cycles (SSSS). Pictures were presented individually on index cards at a 2-s rate and in a new random order on each single cycle. Participants were asked to name each displayed object to ensure that they knew the correct name for the object. When a child was not able to correctly label one of the objects (which happened only very infrequently), the experimenter corrected him or her and the child repeated the correct label. In the test-plus-restudy condition, there were also four successive cycles, but in contrast to the restudy condition, two study cycles were alternated with two test cycles (STST). In these test cycles, participants were asked to orally recall as many of the study items as they could, in any order they wished. Participants' verbal responses during both test cycles were noted by the experimenter.

In both practice conditions, each of the first three cycles was followed by an unrelated 30-s filler task, and, after the final cycle, there was a 5-min distractor task, in which all children were asked to color mandalas. Afterwards, participants completed the final retention test and were given 60 s to recall in any order they wished as many items as possible from the study list. Participants' verbal responses during this test period were noted by the experimenter. One half of the participants completed the test-plus-restudy condition first and the restudy condition second, with a break of several days between conditions; for the other half of the participants, order of conditions was reversed.

### 2.2. Results

**Figure 2:**
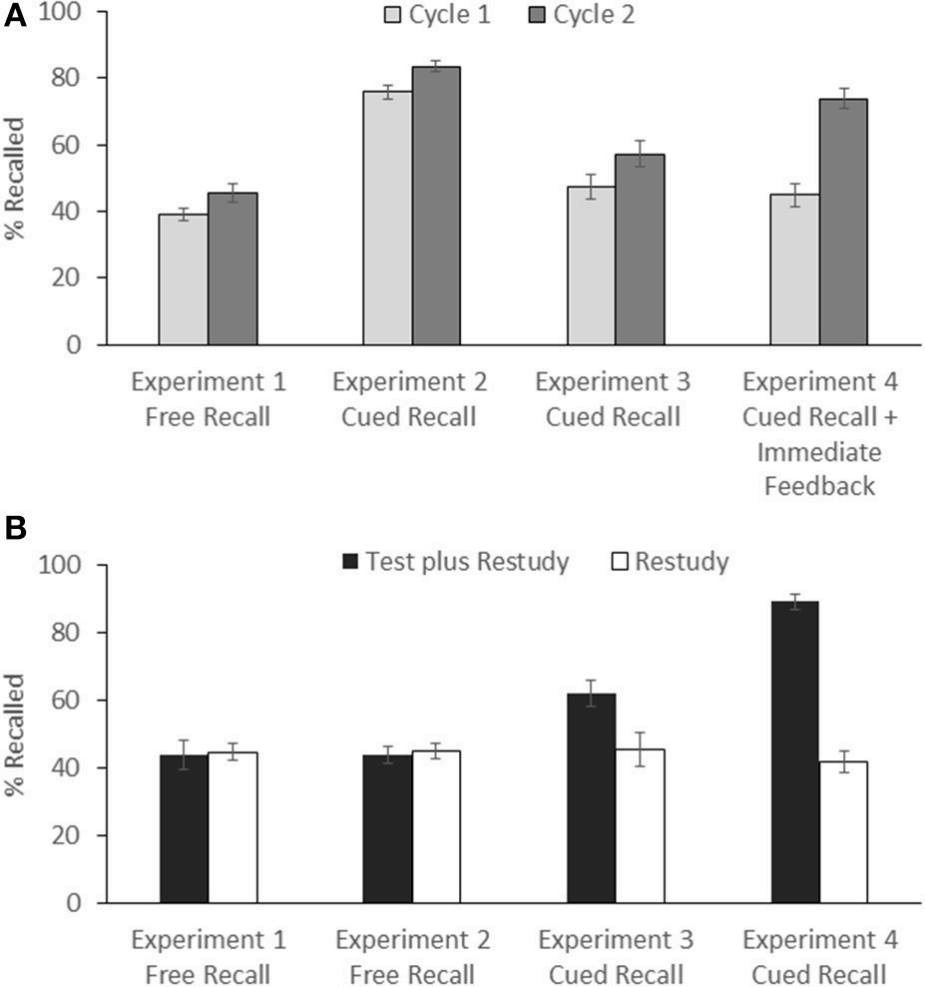
Preschool children's recall performance in Experiments 1–4. **(A)** Mean success rates on the first and second practice cycles in the test-plus-restudy condition. The labels on the X-Axis indicate the practice format. **(B)** Mean performance on the final test as a function of type of practice (test plus restudy vs. restudy). The labels on the X-Axis indicate the test format. Error bars represent standard errors.

Mean success rates were 39.1% during the first retrieval practice cycle, and 45.7% during the second retrieval practice cycle. The difference between cycles was significant, *t*_(31)_ = 2.680, *p* = 0.012, *d* = 0.493 (see Figure 2A). During the final test, the children recalled 43.5% of the items in the retrieval-practice condition and 44.5% of the items in the restudy condition, *t*_(31)_ < 1, suggesting that preschoolers did not benefit from retrieval practice (see Figure 2B).

### 2.3. Discussion

Results from Experiment 1 indicate that preschool children may show no testing effect when free-recall practice and test formats are employed, and when no immediate feedback is provided. The finding thus contrasts with the results from the Fritz et al. (2007) study, which showed a reliable testing effect in preschool children. However, unlike Experiment 1, which employed a relatively demanding retrieval task with free-recall practice and test formats, Fritz et al. employed a less demanding task with cued-recall practice and test formats, and immediate feedback during practice. This difference in settings may account for the contrasting findings. The goal of Experiment 2 thus was to examine whether the difficulty of the retrieval-practice task can influence the testing effect in preschoolers. Indeed, results from Rowland's (2014) meta-analysis suggest that, at least in young adults, the testing effect is typically reduced when success rates during retrieval practice are below 50%. In Experiment 1, mean success rates were well below 50% (around 42%) and Experiment 2 therefore examined whether higher success rates during practice might be the key for obtaining a testing effect in preschoolers.

While Experiment 1 showed no testing effect in preschoolers, Lipowski et al. (2014) reported a reliable testing effect in younger and older elementary school children when a similar testing effect task was employed, which might indicate a developmental trend. However, experimental tasks were not perfectly identical between studies, with a difference in the number of restudy cycles in the original learning phase. In particular, Lipowski et al. included one additional final restudy cycle in both the restudy (SSSSS) and test-plus-restudy (STSTS) conditions. In the test-plus-restudy condition, this restudy cycle might have served as a (delayed) feedback opportunity, which might have contributed to the observed testing effect in school-aged children. Therefore, a further goal of Experiment 2 was to revisit the issue of whether there is a developmental trend in the testing effect, examining the presence of the testing effect in both preschoolers and elementary school children, using the same task for the different age levels.

## 3. Experiment 2

The task empolyed in Experiment 2 was identical to Experiment 1, with the sole exception that, in order to increase success rates during practice cycles, a cued-recall practice format was chosen in Experiment 2. In this cued-recall task, children were presented with pixelated versions of the study items and were asked to correctly name the objects. If higher success rates were sufficient to induce the testing effect in preschoolers with this task, Experiment 2 should show a testing effect in prescholars. Experiment 2 also sought to determine potential developmental trends in the testing effect and, to this end, examined both preschool children and younger and older elementary school children. If both the results of Experiment 1 and of Lipowski et al. (2014), which arose under slightly different experimental conditions, generalized to the present setting, then a larger testing effect may arise for the elementary school children than the prescholars.

### 3.1. Method

Thirty-two preschool children (4–5 years; *M* = 4.7, *SD* = 0.5), 32 younger elementary school children (6–7 years; *M* = 6.7, *SD* = 0.5), and 32 older elementary school children (8–9 years; *M* = 8.3, *SD* = 0.5) took part in the experiment. The preschoolers were recruited from two kindergartens, and the school children from three elementary schools in Regensburg, Germany. All children were tested individually in a quiet room, and were asked to complete a memory task that was identical to the task used in Experiment 1. The only difference between the task employed in Experiment 1 and in Experiment 2 was that during retrieval-practice cycles, blurred versions of each of the study pictures (created with the image editing software Picasa; version 3.9.137) were presented in random order at a 2-s rate and the children were asked to name the pictures. We used slightly more pixelated versions of the pictures for younger and older elementary school children (i.e., each image consisted of 23 × 17 pixels) than for preschool children (i.e., each image consisted of 32 × 24 pixels) to ensure comparable success rates during retrieval practice across age groups (for an example, compare Figure 1B, middle vs. right panel).

### 3.2. Results

Mean success rates across the two practice cycles were 79.7% for the preschoolers, 77.7% for the younger school children, and 79.7% for the older school children. The difference between conditions was not significant, *F*_(2, 93)_ < 1.

**Figure 3:**
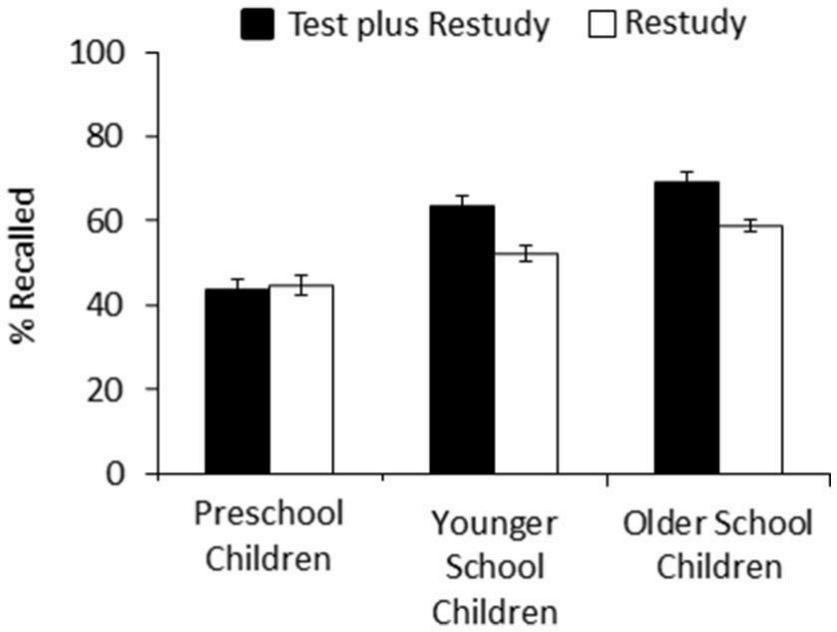
Percentage of correctly recalled items on the final test of Experiment 2, as a function of practice (test plus restudy vs. restudy) and age group (preschool children, younger school children, older school children). Error bars represent standard errors.

A 2 × 3 ANOVA with the factors of practice type (test plus restudy, restudy) and age group (preschoolers, younger school children, older school children) revealed a significant main effect of practice type, *F*_(1, 93)_ = 26.792, *MSE* = 0.01, *p* < 0.001, partial η^2^ = 0.22, reflecting overall higher recall in the test-plus-restudy than the restudy condition. There was also a main effect of age group, *F*_(2, 93)_ = 37.086, *MSE* = 0.02, *p* < 0.001, partial η^2^ = 0.44, indicating an age-related overall increase in recall performance. In addition, a significant interaction between the two factors emerged, *F*_(2, 93)_ = 8.736, *MSE* = 0.01, *p* < 0.001, partial η^2^ = 0.16, suggesting that type of practice affected the three age groups differently. Indeed, planned comparisons showed that there was no reliable difference between the test-plus-restudy and restudy conditions in the preschoolers (43.8 vs. 44.7%), *t*_(31)_ < 1, suggesting that preschoolers did not benefit from retrieval practice, whereas beneficial effects of retrieval practice, compared to restudy, arose for both the younger school children (63.7 vs. 52.3%), *t*_(31)_ = 4.286, *p* < 0.001, *d* = 0.765, and the older school children (69.3 vs. 58.8%), *t*_(31)_ = 5.400, *p* < 0.001, *d* = 1.035 (see Figure 3).

### 3.3. Discussion

The results of Experiment 2 demonstrate a developmental trend for the testing effect. Indeed, while we found beneficial effects of retrieval practice on later free-recall performance in both younger and older elementary school children, retrieval practice did not enhance subsequent free-recall performance in preschoolers. This pattern is consistent with the results reported in Experiment 1 and in Lipowski et al. (2014), which together also suggest a delevelopment trend in the testing effect, although the two lines of findings arose under slightly different experimental conditions,

The finding of Experiment 2 that preschoolers again showed no testing effect, even though success rates during practice were considerably higher than in Experiment 1, again contrasts with the results from the Fritz et al. (2007) study. While there are a few smaller methodological differences between the present Experiment 2 and the Fritz et al. study, the most relevant differences may be that Fritz et al. (i) employed a cued-recall test format and (ii) provided immediate feedback during retrieval practice. Experiments 3 and 4 addressed the potential role of these factors for the testing effect.

## 4. Experiment 3

Experiments 3 and 4 were designed to examine how a cued-recall test format (Experiment 3) and additional immediate feedback during retrieval practice (Experiment 4) affect the testing effect. Because Fritz et al. (2007) employed both a cued-recall test and immediate feedback, we expected that the testing effect should, at least, arise in Experiment 4. Experiment 3 will therefore provide insight into whether a cued-recall test format, in the absence of immediate feedback, can already induce a testing effect in preschool children.

Experiment 3 was identical to Experiment 2, with two differences only. First, we employed a cued-recall task, instead of a free-recall task, on the final test. If using a cued-recall task on the final test is already sufficient to induce a testing effect in preschool children, then a testing effect should arise in Experiment 3. Second, in order to match the number of reexposure cycles for the studied items in the retrieval practice conditions of Experiments 3 and 4, we added a final restudy cycle to both the test-plus-restudy (STSTS) and the restudy (SSSSS) conditions in Experiment 3. In Experiment 4, the two restudy cycles will be replaced by immediate feedback cycles (see below).

### 4.1. Method

Twenty-eight preschoolers (3–6 years; *M* = 4.8, *SD* = 0.5, 16 female) were recruited from two kindergartens in Regensburg, Germany. All children were tested individually in a quiet room. The children were asked to complete the same memory task as in Experiment 2 but, different from Experiment 2, practice cycles consisted of two retrieval-practice cycles and three study cycles (STSTS) in the test-plus-restudy condition, and five study cycles (SSSSS) in the restudy condition. Furthermore, during the final test, participants were presented with blurred versions of each of the study pictures in a random order at a 3-s rate and asked to name the pictures (like in the test-plus-restudy condition). We used slightly more pixelated versions of the pictures in Experiments 3 and 4 during both retrieval practice and the final test than in Experiment 2 to reduce success rates during practice and, therefore, prevent potential ceiling effects on the final test (for an example, see Figure 1B, right panel).

### 4.2. Results

Mean success rates were 47.3% during the first retrieval-practice cycle, and 57.1% during the second retrieval-practice cycle. The difference between cycles was significant, *t*_(31)_ = 5.943, *p* < 0.001, *d* = 1.150 (see Figure 1B). During the final test, the children recalled 62.1% of the items in the retrieval-practice condition and 45.5% of the items in the restudy condition, *t*_(31)_ = 4.452, *p* < 0.001, *d* = 0.878, thus suggesting that preschoolers benefited from retrieval practice (see Figure 2B).

### 4.3. Discussion

Unlike in Experiments 1 and 2, a reliable testing effect arose in Experiment 3. This finding indicates that preschool children can benefit from retrieval practice when a cued-recall test format is applied. The testing effect arose in Experiment 3 although slightly more pixelated versions of the pictures during retrieval practice were used in Experiment 3 than in Experiment 2, resulting in considerably lower mean success rates. This reduction in success rates was intended in order to prevent potential ceiling effects on the final test, again indicating that success rates during practice play a minor role for the size of the testing effect only (see also Experiments 1 and 2).

Although we attribute the difference in results between Experiments 2 and 3 to the difference in test format between experiments, alternatively, one could argue that the difference in results was due to the different number of restudy cycles in the two experiments. Indeed, the final restudy cycle that was added in Experiment 3 might have served as an additional learning opportunity during retrieval practice, thus contributing to the observed testing effect. There are two reasons why we regard this unlikely, however. The one reason is that the results reported in Lipowski et al. (2014) for the younger elementary school children and those reported for younger elementary school children in Experiment 2 are highly similar, both qualitatively and quantitatively. Indeed, although only in the Liposwki et al. study a second restudy cycle was employed, the size of the testing effect was quite similar between studies (10 vs. 12%), and overall recall levels were quite similar as well. The second reason is that preschoolers may not have benefited considerably more from an additional study cycle than school-aged children because children's ability to profit from (delayed) feedback has been shown to increase from preschool to early school years (e.g., Dufresne and Kobasigawa, 1989; Hembacher and Ghetti, 2013). Experiment 4 addresses the issue of whether providing immediate feedback during retrieval practice increases the size of the testing effect in preschool children.

## 5. Experiment 4

Experiment 4 was identical to Experiment 3, with the sole difference that, in the STSTS condition, the two restudy cycles were replaced by immediate feedback during retrieval practice. As a result, Experiment 4 more closely resembled the Fritz et al. (2007) study, which also applied cued-recall practice and test formats and immediate feedback during retrieval practice.

### 5.1. Method

Twenty-eight preschoolers (3–6 years; *M* = 4.9, *SD* = 0.5, 9 female) were recruited from two kindergartens in Regensburg, Germany. All children were tested individually in a quiet room. The children were asked to complete the same memory task as in Experiment 3, with the only difference that, during retrieval practice, participants (i) were shown blurred versions of each of the study pictures at a 3-s rate and asked to name the pictures, and (ii) after responding were shown the intact picture for 2 s as immediate feedback. In the restudy condition, the items were shown five times in successsion (SSSSS), with an unrelated 30-s filler task between cycles.

### 5.2. Results

Mean success rates were 45.0% during the first retrieval-practice cycle, and 73.8% during the second retrieval-practice cycle. The difference between cycles was significant, *t*_(27)_ = 13.023, *p* < 0.001, *d* = 2.532 (see Figure 1B). During the final test, the children recalled 89.1% of the items in the test-plus-restudy condition and 41.8% of the items in the restudy condition, *t*_(27)_ = 15.319, *p* < 0.001, *d* = 2.986, suggesting that the preschoolers showed a pronounced benefit from retrieval practice, relative to restudy practice (see Figure 2B).

### 5.3. Discussion

Results of Experiment 4 replicate the finding of Experiment 3 that preschool children can show a testing effect with a cued-recall test format. This holds while the magnitude of the effect was much more pronounced in Experiment 4 than Experiment 3 (around 47% vs. around 17%), suggesting that immediate feedback during retrieval practice can play a critical role for the size of the testing effect. Interestingly, the magnitude of the testing effect was more pronounced than in the Fritz et al. (2007) study, which reported a testing effect of around 10% in preschool children^1^. Possibly, the discrepancy between studies has to do with the fact that Fritz et al.'s participants were, on average, 4.4 years old, and thus about half a year younger than participants in the present Experiment 4. Indeed, children's ability to benefit from immediate feedback develops with children's age (e.g., Destan et al., 2014). As a whole, the results converge on the view that the testing effect is present in preschool children when a cued-recall test format and immediate feedback during practice are employed.

## 6. General discussion

The findings from the present study demonstrate that preschool children can show the testing effect. In particular, the results of Experiment 4 indicate that a reliable testing effect can arise when cued-recall tasks are employed during both the retrieval-practice and final-test phases, and when, in addition, immediate feedback is provided during retrieval practice. This finding is consistent with the the results of the Fritz et al. (2007) study, which also reported a testing effect in preschool children when cued-recall tasks were employed during the retrieval-practice and final-test phases, and immediate feedback was provided during retrieval practice. Expanding on the Fritz et al. findings, the results from the present Experiment 3 additionally indicate that, in preschoolers, the testing effect can already be induced in the absence of immediate feedback during retrieval practice, even though the effect may be considerably smaller than in its presence (for a discussion, see below).

Besides, the results from the present study suggest that the testing effect does not yet arise as consistently in preschoolers as young adults. For one, cued-recall formats during both retrieval practice and the final test appear to be critical for the presence of the testing effect in preschoolers, as is indicated by the fact that we did not find a testing effect when free-recall formats were employed during both retrieval practice and the final test (Experiment 1), and when a cued-recall format was employed during retrieval practice only (Experiment 2)^2^. Our findings also demonstrate that immediate feedback can amplify the magnitude of the testing effect, because the effect was much more pronounced in the presence (Experiment 4) than the absence (Experiment 3) of such feedback, which mimics results with young adults (Rowland, 2014). However, in the present study immediate feedback was provided only when there was a cued-recall task at test. Future research is therefore required to examine whether immediate feedback can already induce a testing effect in preschoolers when a more demanding free-recall task is applied at test. If so, cued recall during both retrieval practice and at test, as well as immediate feedback during retrieval practice could be considered sufficient, though not necessary, conditions for the existence of the testing effect in preschool children.

Experiment 4 employed immediate feedback during retrieval practice, while in Experiments 1–3, retrieval-practice cycles were followed by restudy cycles. These restudy cycles can also be considered feedback loops, albeit delayed feedback loops. Indeed, in Experiments 1–3, all 16 items of the study list were retrieval practiced during a test-plus-restudy cycle before the whole list was restudied, thus creating a considerably longer delay between the retrieval-practice and feedback opportunities for a given item than in Experiment 4, in which there was no delay at all between retrieval practice and feedback. Our findings thus suggest that preschool children show a larger testing effect with immediate than delayed feedback. Such pattern would contrast with the results from Rowland's (2014) meta-analysis which showed that, in young adults, delayed feedback can be at least as beneficial for the testing effect as immediate feedback. However, prior developmental work suggests that the effects of delayed feedback can rely on metacognitive control processes that may develop during early elementary school years, and may still be mostly absent in younger age groups (e.g., Dufresne and Kobasigawa, 1989; Hembacher and Ghetti, 2013; Destan et al., 2014), which is consistent with the present study showing no clear benefits of delayed feedback in preschool children.

A factor that may have contributed to the testing effects observed in Experiments 3 and 4 is transfer-appropriate processing. That is, the children may have benefited from the match between the retrieval-practice and final-test formats in these experiments (Morris et al., 1977; Kolers and Roediger, 1984). Due to this match, retrieval practice may not only have involved practice of the study items, but also practice of the test format itself, which may have contributed to the presence of the testing effect in the final test. Given that the degraded picture task that was employed during practice and test may have been a novel task that most children will not have encountered before, experience with the task during retrieval practice may have been particularly beneficial. Results of Experiment 1 indeed indicate that transfer-appropriate processing alone was not responsible for the results of Experiments 3 and 4. In this experiment, no testing effect was observed for preschoolers even though there was also a match between the retrieval-practice and final-test formats (which were both free recall formats), suggesting that the novelty of the retrieval task in Experiments 3 and 4 critically contributed to the presence of the testing effect. Future work may examine the issue in more depth, investigating the possible role of the match between retrieval-practice and final-test formats for different types of retrieval tasks.

While our findings thus suggest some role of transfer-appropriate processing for the testing effect in preschoolers, they are not easily reconciled with other explanations of the testing effect in young adults, like retrieval effort and elaborative retrieval. The retrieval-effort hypothesis (Bjork, 1975) assumes that more demanding practice leads to a deeper level of strengthening of the practiced material, which should give rise to a greater testing effect on a later memory test. The elaborative retrieval hypothesis (Carpenter, 2009; Pyc and Rawson, 2010) claims that retrieval practice induces more elaborative processing than restudy does: when attempting to retrieve a target item from memory, semantically related items may be activated while searching for the target information and become linked to the target item. Critically, such extra information may be activated mainly during more difficult retrieval tasks, and may be less activated, or not activated at all, during easier retrieval tasks or restudy opportunities. Thus, both hypotheses lead to the expectation that more difficult practice formats should lead to better long-term retention than easier practice formats, which, however, is not what the present results show. Rather, the finding of no testing effect when a (relatively difficult) free-recall practice format was used (Experiment 1), but a reliable testing effect when a (less demanding) cued-recall practice format was employed (Experiments 3 and 4) suggests that, in preschoolers, moderately difficult practice formats may produce larger memory benefits. This suggestion must remain preliminary, however, because the present experiments were not designed to test accounts of the testing effect directly.

One further study has recently demonstrated that the testing effect can arise in preschool children when a spatial location memory task is employed (Hotta et al., 2017). In this study, the children learned the locations of eight small toys in a partitioned box, before they were either asked to put each of the toys in its place by themselves (test-plus-restudy condition) or they were shown by the experimenter where to put each of the toys (restudy condition). Results showed that the children's memory of the object locations was reliably enhanced in the test-plus-restudy condition, relative to the restudy condition. Critically, like in the Fritz et al. (2007) study and the present Experiment 4, Hotta et al. (2017) applied a task in which immediate feedback was provided during retrieval practice and cues were present during both retrieval practice and at test. The results are thus consistent with the present conclusion that the existence of the testing effect in preschool children largely depends on the presence of cued-recall formats and immediate feedback during practice.

The results of Experiments 2 suggest a developmental trend in the testing effect when using free recall at test. In fact, while we found a reliable testing effect in both younger and older elementary school children, no benefit of retrieval practice arose for preschool children. On the basis of the findings from the present Experiment 1 and the Lipowski et al. (2014) study, the suggestion arises that such developmental trend may also be present with other retrieval practice formats, like free recall. Indeed, using a free recall format during retrieval practice, Experiment 1 showed no testing effect in preschoolers, while Lipowski et al. showed a reliable testing effect with this recall format in elementary school children (see above). Future work may address the issue in more detail, investigating in greater depth whether the indication of a developmental trend is preserved with other final-test formats and, in particular, when feedback is provided during retrieval practice.

From a more practical perspective, the present results suggest that preschool teachers may want to employ learning tasks that include retrieval practice. Such practice can enhance preschoolers' memory for the practiced material, in particular, if immediate feedback is provided. Whether retrieval practice is beneficial for preschoolers, or not, however, seems to depend on procedural details of the practice and testing tasks. Beneficial effects may arise more easily if instructors choose practice tasks that include enough cue support to ensure successful practice of the target information.

### 6.1. Data availability statement

Datasets are available on request: The raw data supporting the conclusions of this manuscript will be made available by the authors, without undue reservation, to any qualified researcher.

## Author contributions

OK was responsible for the conception, data analysis, and interpretation of Experiments 2, 3, and 4, and with drafting the manuscript. MA was responsible for the conception, data analysis, and interpretation of Experiment 1, and gave critical input for various revisions of the manuscript. K-HB was involved with the conception and interpretation of Experiments 1–4. He also helped drafting the manuscript and gave critical input for various revisions of the manuscript.

### Conflict of interest statement

The authors declare that the research was conducted in the absence of any commercial or financial relationships that could be construed as a potential conflict of interest.

## Footnotes

^1^We estimated the size of the effect on the basis of visual inspection of Figure 3 in the Fritz et al. (2007) study.

^2^Recent findings suggest that elementary school children can fail to show a testing effect for complex textbook materials when a free-recall practice format and a cued-recall final-test format are used (Karpicke et al., 2014), suggesting that, even for school-aged children, the presence of a testing effect can depend on methodological detail.
